# Synchronous early primary adenocarcinoma of both rectum and gallbladder. Report of a case

**DOI:** 10.1186/1477-7800-2-19

**Published:** 2005-09-14

**Authors:** Timothy Sakellaridis, Stavros Mathioulakis, Christos Antiochos

**Affiliations:** 1 Surgical Department, 401 General Military Hospital of Athens, Athens, Greece

**Keywords:** Adenocarcinoma of gallbladder, colorectal adenocarcinoma, synchronous primary tumor

## Abstract

**Background:**

Synchronous early primary cancers are rare and in addition synchronous adenocarcinoma of both rectum and gallbladder is extremely rare.

**Case report:**

We report an unusual case of synchronous early primary adenocarcinoma of rectum and gallbladder. The patient was a 72-year-old woman with complaints of bloody stools and constipation. An endoscopy revealed adenocarcinoma of the lower rectum. A through preoperative investigation showed also cholelithiasis. The patient underwent abdominoperineal resection and cholecystectomy. The histopathological diagnosis was well to middle differentiate adenocarcinoma of the gallbladder (T2, N0, M0; stage II) and middle differentiate adenocarcinoma of the rectum (T2, N0, M0; stage II).

**Conclusion:**

For the cases of extracolonic primary cancer associated with colorectal primary carcinoma, Warren and Gates' diagnostic criteria are used. All patients with colorectal carcinoma, should undergo a throughout preoperative examination to exclude the possibility of synchronous early primary cancers.

## Introduction

Synchronous early primary cancers are rare. In recent years multiple primary tumors have been documented more frequently. A review of autopsies of 659 cases of multiple primary tumors showed the colon to be the organ most frequently involved, especially among the aged [1,2].

Colorectal cancer is the third most common cancer in males and females. The cumulative lifetime risk of developing colorectal cancer is about 6%. However, it still accounts for 11% of cancer deaths. The risk of colorectal cancer increases with age. Although carcinoma of the gallbladder is a rare tumor, it is the most common malignancy of the biliary system and the fifth most common cancer of the gastrointestinal tract mostly among patients in their seventh and eighth decades.

We report a case of a female who presented with adenocarcinoma of rectum and cholelithiasis. The histopathology revealed early primary adenocarcinoma of both the rectum and gallbladder.

## Case report

**Figure 1:**
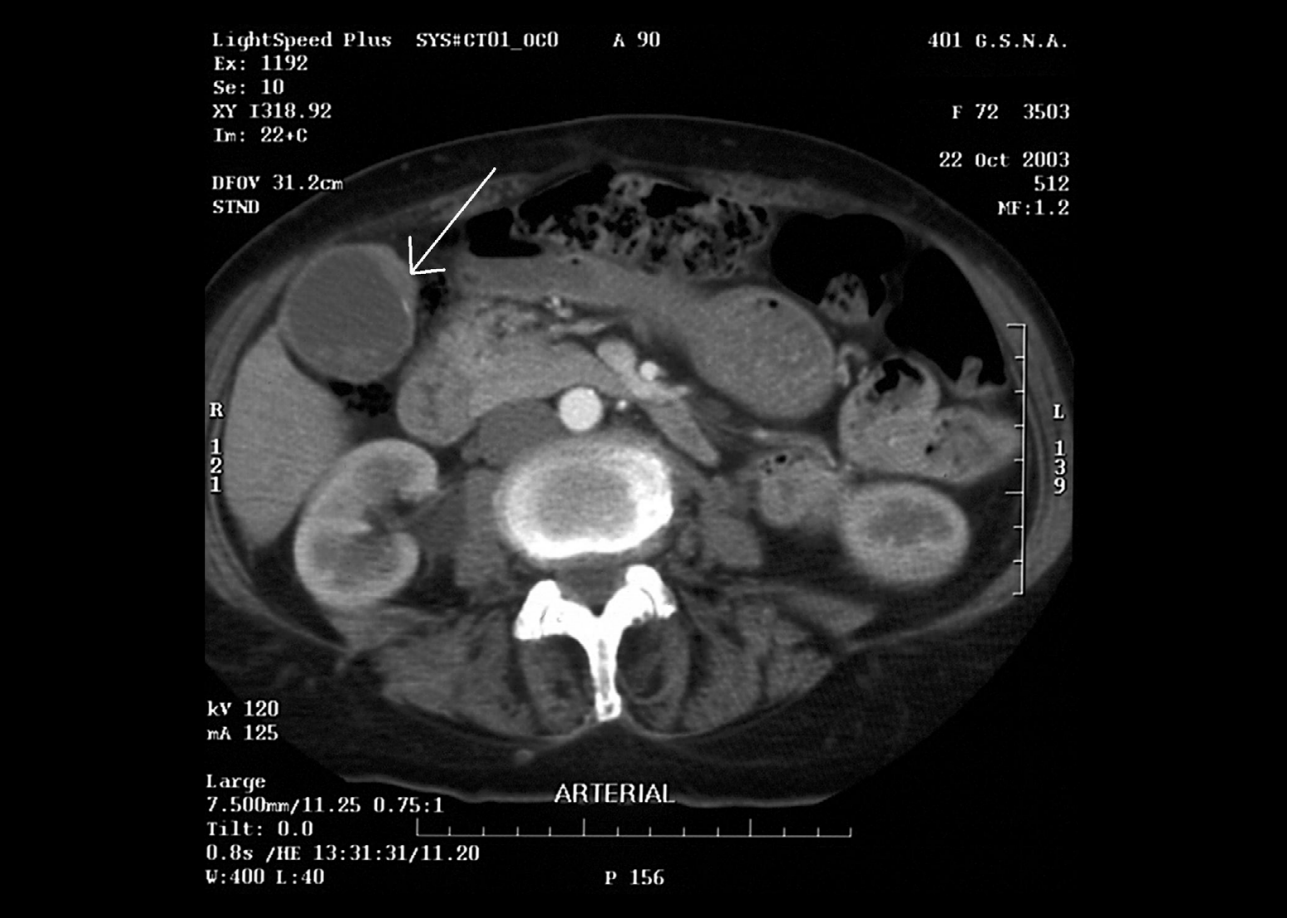
Computed tomography, showing cholelithiasis with thickening of gallbladder anterior wall (arrow in the figure).

A 72-year-old Caucasian woman was complaining of bloody stools and constipation for three months. There was a palpable mass 3–4 cm from the anal verge. A colonoscopy showed a tumor 3 cm from the anal margin with no other indication of multiple synchronous tumors in the colon. Biopsies of the tumor were positive for adenocarcinoma. An investigation with upper gastrointestinal endoscopy, computed tomography of chest and abdomen showed cholelithiasis with thickening of gallbladder anterior wall (arrow in the figure 1). No evidence of metastatic disease documented. Blood examinations showed anemia (Ht: 28,5% and Hb: 9,5 g/dL) and the rest laboratory evaluation was normal. The carcinoembryonic antigen (CEA) was: 15,9 ng/ml (range 0,0–10,0). She had a cholecystectomy and abdominoperineal resection of the rectum. The histopathological diagnosis was a moderately differentiated adenocarcinoma of the gallbladder (T2, N0, M0; stage II) and differentiated moderately adenocarcinoma of the rectum (pT2, N0, M0 in TNM/UICC, stage A of the Dukes staging system and stage B1 of the modified Astler – Coller classification). No adjuvant chemotherapy was required post-operatively. The patient joined a five-year follow up programme and is doing well six months after surgery.

## Discussion

Synchronous adenocarcinoma of both rectum and gallbladder is extremely rare. Review of the pertinent literature revealed no more than five cases worldwide [3-7]. Synchronous cancers are defined as those diagnosed at the same time or within six months; cancers are considered metachronous when the second tumor is diagnosed more than six months after the first^1^. For the cases of extracolonic primary cancer associated with colorectal primary carcinoma, Warren and Gates' diagnostic criteria are used [1]: 1) Each of the tumors have to present a definite picture of malignancy; 2) Each have to be distinct; and 3) The probability that one is metastatic from the other has to be excluded.

All patients with colorectal carcinoma, in addition to the history and physical examination, should undergo a throughout preoperative examination, which should include chest radiograph, complete blood cell count, liver function tests, electrolytes, urinalysis, carcinoembryonic antigen (CEA) determination, fecal occult blood testing (FOBT) (using Hemoccult or Hemoquant), full colonoscopy, double contrast barium enemas, endorectal ultrasound for rectal tumors and abdominopelvic computed tomography scan. In colorectal cancer patients without evidence of distant metastases, a complete meticulous surgical exploration should not be denied. When multiple lesions or evidence of distant metastases are found, the operation should be individualized according to the location, state of spread and the patient's condition. Postoperative follow-up should include a complete blood tests and throughout examination of the remainder colon at least every 6 months. Metachronous cancer may appear as long as 15 years after the first cancer has been removed [1]. Extracolonic primary cancer is reported more frequently in the skin, stomach, breast, urinary bladder and prostate, and it may occur as long as 17 years before, or 20 years after, the diagnosis of the colorectal cancer [1]. Those findings illustrate the pitfalls in assuming any lesion to be a metastasis or a recurrence without pathologic confirmation.

The prognosis of these patients appears to equal or to be only slightly worse than, that for a single colorectal cancer [1]. There have been evidence that in some cases this entity can be ascribed to a genetic defect or an unknown carcinogenic agent or be part of cancer family syndrome [4,8,9].
